# Complete genome sequences of two *Campylobacter bilis* isolates from layer chickens with spotty liver disease

**DOI:** 10.1128/mra.01305-24

**Published:** 2025-05-23

**Authors:** Eman Gadu, Amro Hashish, Mostafa M. S. Shelkamy, Maria Chaves, Mariela E. Srednik, Yuko Sato, Mohamed El-Gazzar

**Affiliations:** 1 Department of Veterinary Diagnostic and Production Animal Medicine, Iowa State University, Ames, Iowa, USA; 2 Department of Avian and Rabbit Diseases, Faculty of Veterinary Medicine, Mansoura University, Mansoura, Egypt; 3 National Laboratory for Veterinary Quality Control on Poultry Production, Animal Health Research Institute, Agriculture Research Center, Giza, Egypt; 4 Department of Avian and Rabbit Medicine, Faculty of Veterinary Medicine, Suez Canal University, Ismailia, Egypt; 5 University of Wyoming Department of Veterinary Sciences, Laramie, Wyoming, USA; Rochester Institute of Technology, Rochester, New York, USA

**Keywords:** spotty liver disease, poultry, *Campylobacter bilis*, genomes

## Abstract

*Campylobacter bilis* has been reported as a second causative agent of spotty liver disease, which leads to liver necrosis, increased mortality, and decreased egg production in laying hens. Here, we present the first two complete genome sequences of *C. bilis* isolates from commercial layer chickens from the United States.

## ANNOUNCEMENT

Spotty liver disease (SLD) is a reemerging acute infectious disease caused by *Campylobacter* species in chickens leading to significant increases in mortality and drops in egg production (1, 2). Although SLD has been observed for several decades, its specific etiology was not confirmed until 2015 (3). Van and colleagues proposed the name *Campylobacter hepaticus* for the bacterial agent and demonstrated its role as a causative agent of SLD (4). Recently, *Campylobacter bilis* has been reported as a second causative agent of SLD (5). However, to date, no complete genome sequences of *C. bilis* have been reported. In this study, we address this gap by presenting the complete genome sequences of two *C. bilis* isolates from bile samples received in Iowa State University Veterinary Diagnostic Laboratory from two individual chickens from the same commercial layer chickens flock affected by SLD. These isolates were initially misidentified as *C. hepaticus* and were later accurately diagnosed as *C. bilis* using conventional PCR assays and Sanger sequencing (6). The identification was further confirmed through whole-genome sequencing with the Illumina MiSeq system (Illumina, San Diego, CA, USA).

The bile samples were directly plated on a 5% sheep blood agar plate under microaerophilic conditions at 42°C for up to 7 days. Afterward, *C. bilis* isolates were grown microaerophilically on blood agar at 37°C for 48–72 h. For Illumina sequencing, DNA was extracted using the MagMAX Pathogen RNA/DNA Kit (Thermo Fisher Scientific, Waltham, MA, USA). The extracted DNA was then used to prepare sequencing libraries with the 500-Cycle v2 Reagent Kit (Illumina, San Diego, CA, USA), generating 250 paired-end reads. The sequencing was performed using an Illumina MiSeq system (Illumina, San Diego, CA, USA). For Oxford Nanopore Technologies (ONT) sequencing, DNA extraction was prepared using the gram-negative bacteria high molecular weight DNA extraction protocol QIAGEN Genomic-tip 20/GDNA Kit (QIAGEN, GERMANY). Nanopore libraries were prepared using the Ligation Sequencing (SQK-LSK109) and native barcoding (EXP-NBD104) kits according to the manufacturer’s protocol (ONT, Oxford, UK). The library was loaded onto a FLO-MIN106 R9.4.1 flow cell and sequenced with the MinION Mk1B (ONT, Oxford, UK) for 72 hours. Base calling after the run was done using Guppy (v6.5.7) with the super high accuracy mode activated.

**Fig 1:**
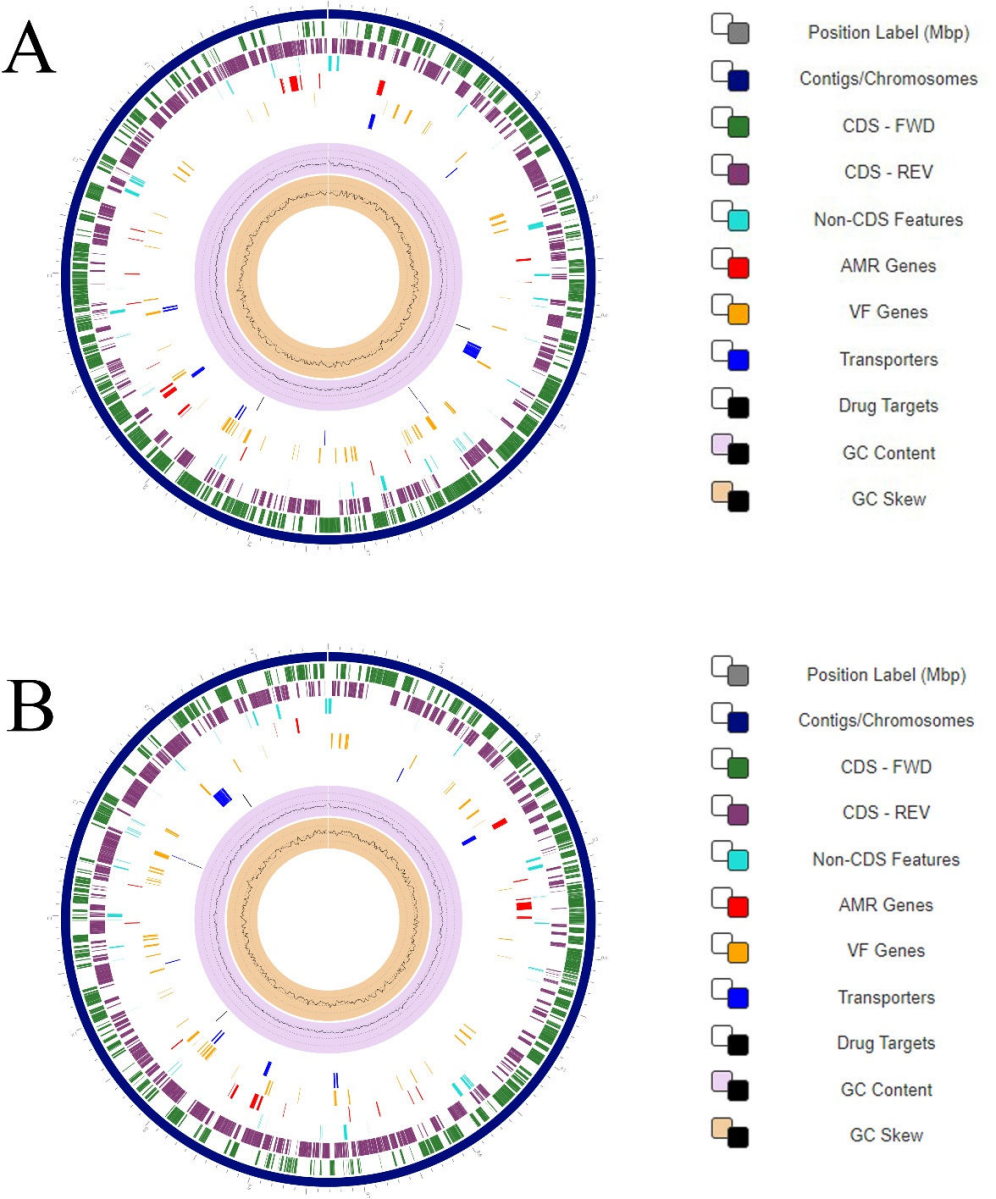
Circle maps of *C. bilis* 64 (A) and *C. bilis* 63 (B) genomes. The outermost to innermost rings of the maps represent the following: (i) the contigs; (ii) coding sequence (CDS) on the forward strand; (iii) CDS on the reverse strand; (iv) RNA genes; (v) CDS with homology to known antimicrobial resistance genes; (vi) CDS with homology to known virulence factors; (vii) GC content and GC skew.

The quality-filtered reads were assembled and rotated using Flye v2.9.1 (7) within the Bacterial and Viral Bioinformatics Resource Center website using the default parameters (8) followed by two rounds of polishing with Illumina reads using Pilon (9). The circularity of the chromosomes was determined based on the Bandage plots (10), and the quality of the assembly was assessed by Quast v5.2 (11). The complete bacterial genome consists of two circular chromosomes (1,461,029  bp and 1,461,042  bp) with an average GC content of 30.8% as shown in Fig. 1. All genomes were annotated using the Genome Annotation tool within the Bacterial and Viral Bioinformatics Resource Center website using the RAST tool kit (RASTtk) for bacteria (12). A summary of the metadata, generated sequences, assembly statistics, and annotation of the genomes is presented in Table 1, and circular genomic maps are presented in Fig. 1. The availability of these genomes will help improve diagnostic capabilities and provide deeper insights into the pathogenicity of *C. bilis* in chickens.

**TABLE 1:** Data associated with the two sequenced *C. bilis* isolates showing metadata, reads generated by each sequencing platform, assembly statistics, annotation of the genomes, sequence typing, average nucleotide identity, and GenBank accession numbers

Parameter	Sequenced *C. bilis* isolates	
	64	63
Metadata		
Species (type of production)	*Gallus gallusdomesticus* (laying hen)	*Gallus gallusdomesticus* (laying hen)
Age of the flock	Not available	Not available
Country	USA	USA
Date of sample collection	1/8/2019	1/8/2019
Isolation source	Bile	Bile
Raw sequencing reads		
Illumina MiSeq paired-end read length (bp)	250	250
Number of Illumina MiSeq reads	896,404	809,700
Average Illumina MiSeq coverage (×)	82.42	76.41
Total Illumina MiSeq sequencing data (Mbp)	137.62	126.34
Number of nanopore reads	436,715	344,074
ONT read N50 (bp)	9,700	10,864
Average nanopore coverage (×)	201.395	198.331
Total Illumina nanopore sequencing data (Mbp)	2,456.92	2,101.14
No. of contigs	1	1
Total length of the chromosome (bp)	1,461,029	1,461,042
Assembly statistics		
GC content (%)	30.80	30.80
N50 for the hybrid assembly (bp)	1,461,029	1,461,042
Number of CDSs	1,567	1,557
Annotation results		
Number of tRNAs	43	43
Number of rRNAs	9	9
Overall genome-related indices		
ANI^*a*^ to NCBI *C. bilis* type strain ASM1680669v1	99.98%	99.99%
dDDH^*b*^ to NCBI *C. bilis* type strain ASM1680669v1	100%	100%
GenBank data		
BioSample accession number	SAMN42738858	SAMN42738857
BioProject accession number	PRJNA1138730	PRJNA1138730
Genome assembly accession number	Chromosome CP168724.1	Chromosome CP168723.1

^*a*^ Average nucleotide identity percentage was calculated via JSpeciesWS online service https://jspecies.ribohost.com/jspeciesws/#home.

^*b*^ Calculated using the online tool available through the GGDC website at https://ggdc.dsmz.de/ “formula 2.”

## Data Availability

The genomes are available from NCBI BioProject number PRJNA1138730 and SRA accessions SRR30006384, SRR30006385, SRR30027102, and SRR30027103 (Table 1).
